# Supplementary material for: Persistence on oral pre-exposure prophylaxis (PrEP) among female sex workers in eThekwini, South Africa, 2016–2020
Source: PLoS One. 2022 Mar 15;17(3):e0265434. doi: 10.1371/journal.pone.0265434 (PMC8923438; doi:10.1371/journal.pone.0265434)
Supplement: S1 Appendix — (DOCX) [file pone.0265434.s001.docx]

**APPENDIX.** Kaplan-Meier survivor function with time-to-event outcome defined as not being retained on PrEP among 2776 female sex workers initiating PrEP through TB HIV Care in Durban, South Africa 2016-2020

| **Month** | **At risk** | **Not retained^+^** | **Net lost^§^** | **Survivor function** | **95% Confidence Interval** |
| --- | --- | --- | --- | --- | --- |
| 1 | 2776 | 1299 | 0 | 0.53 | 0.51-0.55 |
| 2 | 1476 | 73 | 90 | 0.51 | 0.49-0.52 |
| 3 | 1313 | 47 | 178 | 0.49 | 0.47-0.51 |
| 4 | 1088 | 346 | 39 | 0.33 | 0.31-0.35 |
| 5 | 703 | 154 | 42 | 0.26 | 0.24-0.28 |
| 6 | 507 | 25 | 38 | 0.25 | 0.23-0.26 |
| 7 | 444 | 126 | 41 | 0.18 | 0.16-0.19 |
| 8 | 277 | 53 | 2 | 0.14 | 0.13-0.16 |
| 9 | 222 | 22 | 5 | 0.13 | 0.11-0.14 |
| 10 | 195 | 42 | 1 | 0.10 | 0.09-0.12 |
| 11 | 152 | 23 | 3 | 0.09 | 0.07-0.10 |
| 12 | 126 | 0 | 126 | 0.09 | 0.07-0.10 |

^+^Not retained= did not return, client discontinued, provider discontinued

**^§^**Net lost=Lost from follow-up with no outcome determined. No late entries.
