# Supplementary material for: Persistence on oral pre-exposure prophylaxis (PrEP) among female sex workers in eThekwini, South Africa, 2016–2020
Source: PLoS One. 2022 Mar 15;17(3):e0265434. doi: 10.1371/journal.pone.0265434 (PMC8923438; doi:10.1371/journal.pone.0265434)
Supplement: Attachment — Submitted filename: Plos1reviewSep2021final.docx [file pone.0265434.s002.docx]

Persistence on oral pre-exposure prophylaxis (PrEP) among female sex workers in

eThekwini, South Africa, 2016-2020

Review Sep 2021

PLOSone

**INTRO:**

1) PrEP persistence instead of persistence on PrEP sounds better and less clumsy
2) Police arrest seems redundant
3) Reference 20 seems to be the wrong reference for line 115. Also the roll out happened with sex workers first - <https://www.prepwatch.org/early-lessons-south-africa-rollout/>

**METHODS:**

4) When you looked at PrEP initiation were these all new initiations or did some people stop and then initiate again? Please clarify in the methods how your cohort is defined. Results indicate that some were restarts.
5) There are different ways to define PrEP persistence – you could reference how other people have defined this and why you chose your definition. Please also explain why people had to miss 2 consecutive visits to be defined as “discontinued” It seems to be a more generous definition of persistence and may explain why your persistence is higher than other studies
6) Did you bring your participants back for monthly visits in your program? This is not comparable to most standard of care that would see PrEP clients after a month and then 3 monthly. Please describe the program in the methods and justify why this is “real world implementation” (line 118). Please also discuss how many FSW accessed the program in total year on year and how many of the non infected FSW refused to take PrEP. It is important to have context to be able to understand what the results mean.
7) Some results are reported as “significant” please define in the methods what you mean by this

**RESULTS:**

8) General comment on the results is that there are no denominators so it is very difficult to interpret your % results.
9) PrEP initiations increased year on year – please include denominators to understand if the project was scaling up or if you were seeing the same numbers of people and more were taking up PrEP. So something like of the xxx FSW eligible to receive PrEP yyy initiated PrEP per year.
10) Please give the n/N for the PrEP persistence figures – need to see the denominators to be able to interpret your results.
11) PrEP persistence was 53% (95% CI: 51%-55%) 1 month after initiation, 33% (95% CI: 31%-35%) 4-months after initiation, and 18% (95% CI: 7-months after initiation. Is this overall? (line 162)
12) Please clarify how you know your results are significant? What statistical test did you use?

**DISCUSSION:**

13) No limitations mentioned

**FIGURES AND LEGENDS**

14) Figure 1 legend is incomplete
